# Dietary and Gut Microbiota Polyamines in Obesity- and Age-Related Diseases

**DOI:** 10.3389/fnut.2019.00024

**Published:** 2019-03-14

**Authors:** Bruno Ramos-Molina, Maria Isabel Queipo-Ortuño, Ana Lambertos, Francisco J. Tinahones, Rafael Peñafiel

**Affiliations:** ^1^Department of Endocrinology and Nutrition, Virgen de la Victoria University Hospital, Institute of Biomedical Research of Malaga, University and Malaga, Malaga, Spain; ^2^CIBER Physiopathology of Obesity and Nutrition (CIBERobn), Institute of Health Carlos III (ISCIII), Madrid, Spain; ^3^Department of Medical Oncology, Virgen de la Victoria University Hospital, Institute of Biomedical Research of Malaga, University and Malaga, Malaga, Spain; ^4^Department of Biochemistry and Molecular Biology B and Immunology, Faculty of Medicine, University of Murcia, Murcia, Spain; ^5^Biomedical Research Institute of Murcia (IMIB), Murcia, Spain

**Keywords:** polyamines, diet, gut microbiota, metabolism, aging, obesity

## Abstract

The polyamines putrescine, spermidine, and spermine are widely distributed polycationic compounds essential for cellular functions. Intracellular polyamine pools are tightly regulated by a complex regulatory mechanism involving *de novo* biosynthesis, catabolism, and transport across the plasma membrane. In mammals, both the production of polyamines and their uptake from the extracellular space are controlled by a set of proteins named antizymes and antizyme inhibitors. Dysregulation of polyamine levels has been implicated in a variety of human pathologies, especially cancer. Additionally, decreases in the intracellular and circulating polyamine levels during aging have been reported. The differences in the polyamine content existing among tissues are mainly due to the endogenous polyamine metabolism. In addition, a part of the tissue polyamines has its origin in the diet or their production by the intestinal microbiome. Emerging evidence has suggested that exogenous polyamines (either orally administrated or synthetized by the gut microbiota) are able to induce longevity in mice, and that spermidine supplementation exerts cardioprotective effects in animal models. Furthermore, the administration of either spermidine or spermine has been shown to be effective for improving glucose homeostasis and insulin sensitivity and reducing adiposity and hepatic fat accumulation in diet-induced obesity mouse models. The exogenous addition of agmatine, a cationic molecule produced through arginine decarboxylation by bacteria and plants, also exerts significant effects on glucose metabolism in obese models, as well as cardioprotective effects. In this review, we will discuss some aspects of polyamine metabolism and transport, how diet can affect circulating and local polyamine levels, and how the modulation of either polyamine intake or polyamine production by gut microbiota can be used for potential therapeutic purposes.

## Introduction

**Figure 1:**
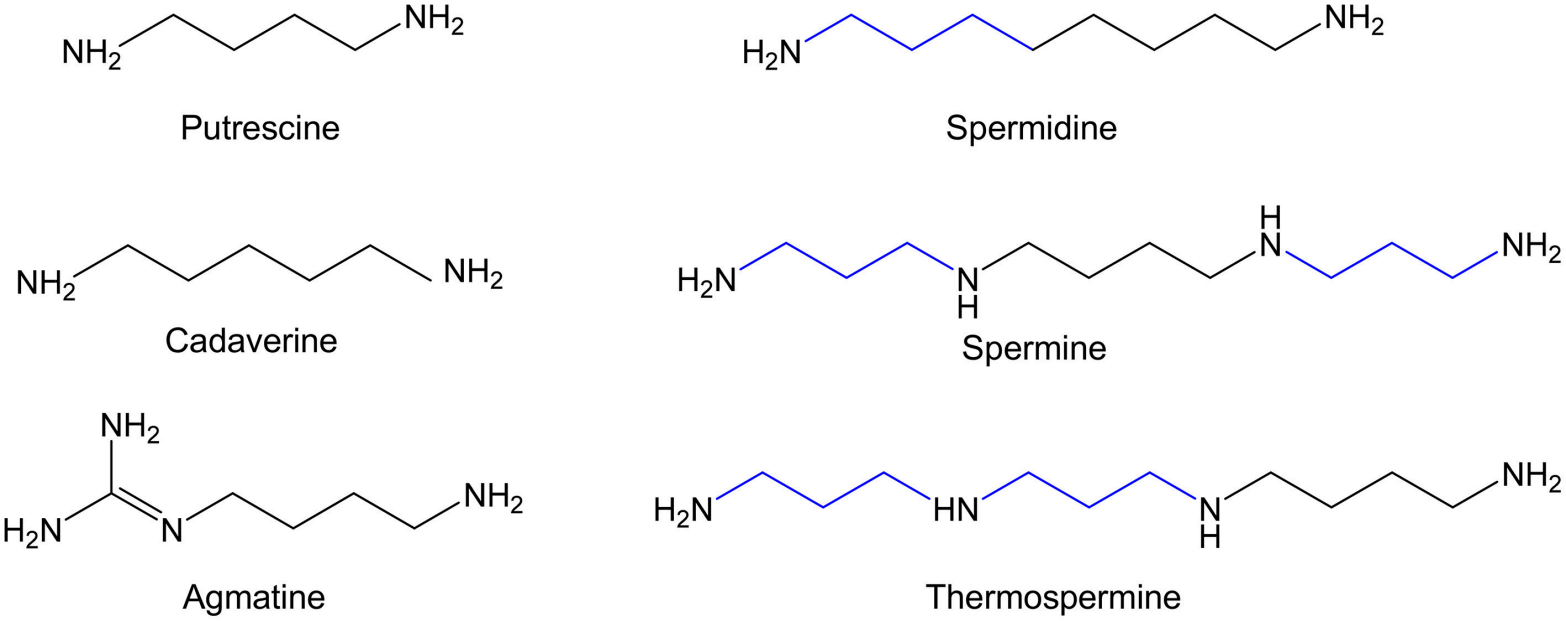
Chemical structure of the major biogenic polyamines.

Polyamines are small aliphatic amines that are present in all living organisms from bacteria to human beings. They can act as polycations since they are positively charged at physiological pH. The major polyamines in mammalian cells are spermidine, spermine, and their precursor the diamine putrescine (1, 2) (Figure 1). In addition of these molecules, microorganisms, and plants also synthesize other types of polyamines such as cadaverine, agmatine, and thermospermine (3–5) (Figure 1), and even minority polyamines (thermine, caldine, thermospermine, etc.) have been detected in extreme microorganisms (6). Agmatine and cadaverine are also present in very low amount in certain mammalian tissues (7, 8). Although polyamines were discovered in the seventeenth century, major advances in their metabolism and functions were achieved in the second part of the last century (2, 9–11). Numerous experiments have shown that due to their polycationic nature, polyamines can readily bind to negatively charged biomolecules including DNA, RNA, proteins, and phospholipids, modulating in many cases the function of these macromolecules (12, 13). When polyamine metabolism was pharmacologically or genetically altered, many relevant biochemical, and cellular processes resulted affected, including translation, transcription, signal transduction, cell proliferation, and differentiation, apoptosis, or cell stress response (14–19). However, the molecular mechanisms by which polyamines exert their biological effects are only partially understood (20).

In humans, only two genetic diseases affecting enzymes of the biosynthetic pathway of polyamines have been described so far (21, 22). However, several lines of evidence have implicated a dysregulation of the polyamine system in hyperproliferative and neurodegenerative diseases (23–27). The alteration of polyamine metabolism in several types of cancer has sustained the interest in using the polyamine pathway as a target for anticancer therapy (28, 29). In this regard, it is clear that the limitation of the cellular polyamine content has a potential interest in cancer chemoprevention or in the treatment of other pathologies such as certain infectious diseases and type 1 diabetes (28, 30, 31). On the other hand, experiments conducted in different cellular and animal models have revealed that the exogenous administration of spermidine or other natural polyamines may exert beneficial effects by affecting processes such as cellular stress, chronic inflammation, or dysregulated lipid or glucose metabolism (32–34). In this review, we will discuss different aspects related with the homeostasis of polyamines in mammalian tissues, including the relevance of the polyamines generated by the gut microbiota, how the endogenous levels of polyamines can be affected by the administration of exogenous polyamines, and the impact that these treatments may exert on the evolution of the biochemical changes associated with aging or obesity-related metabolic diseases.

## Polyamine Functions

Polyamines are essential for life. In fact, the inactivation of the genes that control the biosynthesis of putrescine or spermidine are embryo-lethal in mice (35, 36). This is not surprising due to the multiple essential cellular actions that have being ascribed to polyamines. Thus, owing to their general interaction with nucleic acids, they can affect many processes in which DNA, RNA, or proteins participate as substrates (13, 37, 38). Particularly interesting is the relationship between polyamines and reactive oxygen species (ROS). Although polyamine catabolism may generate potentially toxic products such as H_2_O_2_ and polyamine derived aldehydes associated to pathophysiological consequences (39, 40), numerous *in vitro* and *in vivo* experiments have suggested that spermine and spermidine may act as scavengers of ROS, and then protecting DNA from oxidative damage (41–43). This double-edged role of polyamines appears to be dependent of certain factors (44). One of these factors in *in vitro* studies could be the use of animal serum in the cell culture medium, which contains amino oxidases that can oxidize exogenously administrated polyamines and generate ROS, resulting in cell toxicity independently of the action of the polyamine itself. Interestingly, a recent work demonstrated that in the presence of human serum, polyamine administration to the culture medium does not increase ROS production and does not affect cell viability as in the case of the same experiment in presence of either bovine or horse serum (45). Importantly, studies showing a polyamine-dependent cell toxicity in human cell lines in presence of significant amounts of bovine/horse serum should be reevaluated with human serum to corroborate that toxicity could be due to the production of oxidized polyamine-derived products by the action of serum polyamine oxidases and not to a toxic effect of the polyamines *per se*.

## Polyamine Content and its Regulation

### Cellular Polyamine Levels and Distribution

In general, spermidine and spermine are the most abundant polyamines in mammalian cells. In addition, their absolute values and the spermidine/spermine ratio depend on the type of cell and tissue (46, 47), and their tissue concentrations are affected by several factors including age (46–49). In mammalian cells, polyamines are present at relatively high concentration compared with other biogenic amines. Total polyamine concentration is at the mM range. However, it has been estimated that the free intracellular concentration of each polyamine is much lower (7–15% of total for spermidine, and 2–5% for spermine), due to the fact that a high percentage of total polyamines are bound by ionic interactions to nucleic acids, proteins, and other negatively charged molecules in the cell (12, 50). According to these data, only a small fraction of the cellular polyamines appears to be metabolically active. In addition, another unsettled aspect of polyamines is related to the subcellular distribution of these molecules inside mammalian cells. In rat liver mitochondria, spermidine, and spermine are present at estimated concentrations of 1.21 and 0.62 mM, respectively (51). Polyamines are also stored in vesicles of secretory cells and neurons (52–54). The intracellular levels of polyamines depend on several factors: polyamine biosynthesis, catabolism, uptake, and efflux. It is generally accepted that a high proportion of the cellular polyamines have an endogenous origin, although in some cases the exogenous supply (i.e., dietary and gut microbiota-derived polyamines) may also significantly affect the polyamine content. Regarding circulating polyamines, several studies have shown that they are present at the low μM range in the blood of both humans and mice (55, 56). It is not clear whether blood polyamine levels are age-dependent (48).

### Polyamine Biosynthesis and Catabolism

**Figure 2:**
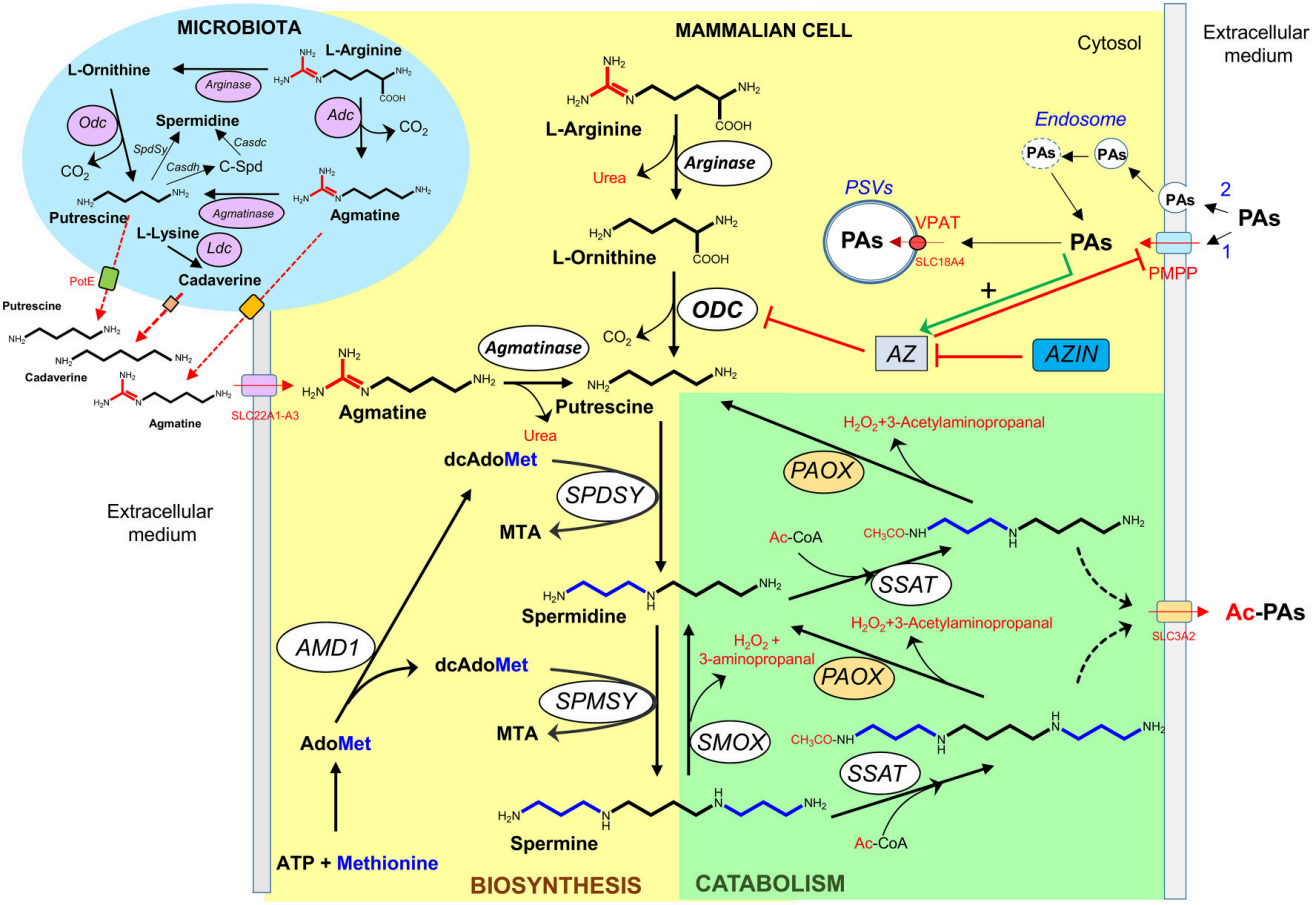
Polyamine metabolism and transport. Biosynthetic (yellow) and degradative (green) pathways of polyamines in mammalian cells, and bacteria (blue). PAs, polyamines; Ac-PAs, acetylated polyamines; C-Spd, carboxyspermidine; PSVs, polyamine sequestering vesicles; PMPP, plasma membrane polyamine permease; VPAT, vesicular polyamine transporter; ODC, Odc, ornithine decarboxylase; Adc, arginine decarboxylase; Ldc, lysine decarboxylase; AMD1, S-adenosylmethionine decarboxylase; SPDSY, Spdsy, spermidine synthase; SPMSY, spermine synthase; SSAT, spermidine/spermine acetyl transferase; PAOX, acetylpolyamine oxidase (microsomal); SMOX, spermine oxidase; AZ, antizyme; AZIN, antizyme inhibitor; Casdh, carboxyspermidine dehydrogenase; Casdc, carboxyspermidine decarboxylase; AdoMet, S-adenosylmethionine; dcAdoMet, decarboxylated S-adenosylmethionine; MTA, methylthioadenosine; Ac-CoA, acetyl-coenzyme A.

In mammalian cells, the polyamine biosynthetic pathway consists in four steps catalyzed by enzymes mainly located in the cytosol (Figure 2). In this process, L-ornithine, and S-adenosyl methionine (AdoMet) are used as substrates. The diamine putrescine is synthesized by ornithine decarboxylase (ODC), a key enzyme that presents a complex regulation (57). This diamine can be converted into spermidine by the addition of an aminopropyl moiety donated by decarboxylated S-adenosyl methionine (dcAdoMet) through the action of spermidine synthase (5, 58). The tetra-amine spermine is synthesized by another aminopropyl transferase (spermine synthase) through the incorporation of another aminopropyl group to the amino butyl end of spermidine (5, 59). The second key-rate enzyme in the synthesis of spermidine and spermine is S-adenosyl methionine decarboxylase (AdoMetDC, AMD1), which produces dcAdoMet used as aminopropyl donor for the formation of both polyamines (60). Other biochemical route that participates in the regulation of the intracellular levels of polyamines is the so-called back-conversion pathway of polyamines (Figure 2). Spermidine/spermine N1 acetyltransferase (SSAT) is a cytosolic enzyme that plays a key role in reducing intracellular polyamine levels, through the acetylation of spermine and spermidine to produce in first instance acetyl-spermine or acetyl-spermidine (61, 62), which are either excreted from the cell or oxidized to spermidine or putrescine, respectively, by an acetyl polyamine oxidase (PAOX) (63). PAOX is a FAD-dependent enzyme located in peroxisomes that generates, apart from the corresponding polyamine, the potentially toxic byproducts 3-acetamidopropanal and H_2_O_2_ (40). Spermine oxidase (SMOX), another FAD-containing enzyme, is capable of directly oxidizing spermine to spermidine, producing a 3-aminopropanal and H_2_O_2_ (64, 65). Due to its localization outside of peroxisomes, the overexpression of the enzyme may enhance the oxidative damage by both increasing H_2_O_2_ production and decreasing spermine levels (40, 66).

Agmatine (decarboxylated arginine) is an aminoguanidine structurally related to polyamines that behaves as a dication at physiological pH. Although agmatine is synthesized in bacteria and plants by arginine decarboxylase (ADC) (Figure 2), its biosynthesis in mammalian cells is controversial. In fact, the product of the initially reported ADC clone (67) was later demonstrated to be devoid of ADC activity by various independent groups. Instead, it encoded an ornithine decarboxylase antizyme inhibitor (AZIN2) (68, 69). Nowadays, multiple pharmacological effects of agmatine have been reported with potential therapeutic interest (70, 71), but to our knowledge clinical applications have not been yet implemented.

### Polyamine Transport

Polyamines can enter and exit the cells through different type of carriers. Whereas, in bacteria and fungi, multiple polyamine transporters have been fully characterized (72), in mammalian cells lesser is known about the molecular components related with polyamine transport (73). It is generally accepted that in these cells the polyamine uptake activity is affected by the polyamine requirements of the cells. Thus, enhanced polyamine uptake is characteristic of cells with either high proliferating activity or in which cellular polyamine levels have been depleted by inhibitors of the biosynthetic pathway (74). Although the kinetics and some biochemical properties of polyamine transport have been studied in different types of cell lines and cell mutants, many questions about the “polyamine transport system” are still unsolved. It is possible that the polyamine carriers belong to the solute transport family (SLC) that contains about 400 annotated members. Different experiments of cell transfection by genes of the organic cation transporter (OCT) family or the cationic amino acid transporter (CAT) family have identified genes that could participate in the polyamine uptake system, but not a clear picture has emerged from all these studies (73, 75–78). On the other hand, polyamine export has also been detected in a variety of mammalian cells, having been identified SLC3A2 as a component of a diamine exporter that could participate in the excretion of putrescine and acetylated spermidine (79) (Figure 2). Apart from the polyamine transport system, endocytic pathways for the uptake of circulating polyamines have been described. In one model, polyamines bind electrostatically to the heparan sulfate chains of glypican-1, a proteoglycan of the cell surface, and then are taken into the cells through endocytosis (80). In another model, polyamines interact with non-defined polyamine binding protein(s) and the complex is internalized by caveolar endocytosis by a process that is negatively regulated by caveolin-1 (81).

### Regulation of Polyamine Levels by Antizymes and Antizyme Inhibitors

The control of the polyamine homeostasis in mammals is crucial for maintaining cellular functions, and dysregulation of the cellular polyamine levels has been associated to diverse pathological conditions (20). Antizymes (AZs) are regulatory proteins involved in the control of intracellular polyamine levels through the modulation of both polyamine biosynthesis and uptake. AZs affect the polyamine biosynthetic route by interacting with the ODC monomer and preventing the formation of active ODC homodimers, and by stimulating the degradation of ODC by the proteasome without ubiquitination (82). They also affect the import of extracellular polyamines by inhibiting the plasma membrane polyamine transport system by a still unknown mechanism (83).

The negative effects that AZs exert on both the biosynthesis of intracellular polyamines and the polyamine uptake can be abrogated by the action of antizyme inhibitors (AZINs), proteins homologous to ODC but devoid of enzymatic activity. AZINs are able to bind AZs even more efficiently than ODC, releasing ODC from the inhibitory ODC-AZ complex (84, 85). AZINs are also able to enhance the uptake of extracellular polyamines, likely by negating the inhibitory action of AZs on the polyamine transport system (86). In addition, AZs and AZINs can also modulate the uptake of agmatine by mammalian cells (87).

## Absorption of Polyamines in the Gastrointestinal Tract

The polyamines present in the lumen of the gastrointestinal tract may have different origin: food, intestinal microbiota, pancreatic-biliary secretions, and intestinal death cells. Although a precise quantitation of the contribution of each process to the whole polyamine pool is non-existent, it is believed that dietary polyamines is the major source of luminal polyamines in humans and animals (88, 89). Polyamine levels have been analyzed in hundreds of food items by different groups (49, 88, 90–93). In general, fruits and cheese are rich in putrescine, whereas vegetables and meat products contain high levels of spermidine and spermine, respectively (94). Despite the high variability of the polyamine content in foods, it has been estimated that a standard human diet provides hundreds of micromoles of polyamines per day (88), with small differences between the major polyamines when it is calculated from diets from different countries (95).

The polyamine levels in the intestinal lumen change over time after a meal, from millimolar levels immediately after the ingestion, to much lower values in the fasting period (96). Luminal polyamines can be taken up mainly by the small intestine. Experiments using radiolabeled polyamines administered intragastrically to rats showed that polyamines can be absorbed and distributed to different tissues (88). This distribution was not uniform, since polyamines were accumulated preferentially in those tissues stimulated to proliferate (88). The first barrier for the uptake of polyamines from the intestinal lumen is formed by the epithelial cells of the intestinal mucosa. In isolated enterocytes, as in other mammalian cells in culture, polyamines can be taken up by different specific polyamine transporters, as already commented in the polyamine transport section. The *in vivo* polyamine uptake by the intestinal cells is more complex due to the existence of different polyamine transporters in the apical and basolateral membranes, as shown by studies using brush-border and basolateral membrane vesicles of the enterocyte (97). According with experimental data, luminal polyamines could be taken by enterocytes by transport across the apical membrane and extruded across the basolateral membrane by low affinity transporters to the systemic circulation (96). It was also hypothesized that the majority of luminal polyamines could be passively absorbed via the paracellular route (96). Whereas, most of spermidine and spermine taken up by the intestinal cells are not metabolized in these cells, a variable proportion of putrescine is transformed into other compounds including spermidine, γ-aminobutyric acid (GABA) and succinate (88, 98). In the small intestine of rats, putrescine can be transformed into succinate acting as a source of instant energy (99). The absorption of polyamines appears to be rapid, since experiments using an *ex vivo* rat model revealed that values about 70% of the ^14^C-polyamines administered to the jejunal lumen were found in the portal vein, after 10 min of polyamine administration (100). Most of the studies on luminal polyamine uptake and their distribution through the body have been based on the acute administration of a low dose of labeled polyamines to rats. Recently, as described in other section, many studies have reported beneficial effects of a prolonged oral administration of either spermidine or spermine to rodents (101–104). However, in most studies tissue polyamine levels were not reported. In mouse models, prolonged administration of polyamine-rich diets have been seen to increase blood levels of spermidine and/or spermine (56, 105, 106). In aged mice spermidine levels significantly increased in blood (107) and liver (101) after supplementation of the drinking water with 3 mM spermidine for 6 months. In line with this, a 28-day oral supplementation of adult mice with 50 mg/kg of spermidine resulted in a significant increase of spermidine in whole blood and heart (but not in brain) of females, but not in males (106). In humans it has been shown that a prolonged intake (2 months) of polyamine-rich products such as natto (fermented soy) produces a significant rise in the levels of spermine (but not spermidine) in blood (56). More recently, the results of a clinical trial using spermidine supplements in older human subjects have been reported, showing no differences in blood polyamine levels between controls and spermidine-supplemented individuals at 3 months of follow-up (106)s.

A part of the absorbed luminal polyamines remains in the intestinal cells. This is not surprising since the intestinal tissue is one of the most rapidly proliferating tissues, and in general the polyamine uptake of intestinal cells is associated with the proliferative stage. Dietary polyamines might be important for the development of the digestive tract, and also for the maintenance of the adult digestive tract (108). Moreover, polyamine reservoirs are not only responsible for maintaining the rapid turnover and high proliferation rates of the intestinal epithelial cells but also for enhancing the integrity of the intestinal barrier. Thus, polyamines are able to stimulate the production of intercellular junction proteins, such as occludin, zonula occludens 1, and E-cadherin, which are essentials to regulate the paracellular permeability and reinforcing epithelial barrier function (109).

Furthermore, intestinal polyamine pools are necessary for the postnatal development of the gastrointestinal tract. These data were confirmed in a study using pup rats, where the polyamine administration was able to induce the production of mucus and secretory IgA in the small intestine, while rats fed with a polyamine-deficient diet developed intestinal mucosal hypoplasia (110). In addition, the oral administration of polyamines to suckling rats accelerated the maturation process affecting intestine, liver and pancreas. Interestingly, the precocious maturation of the intestine only was achieved when spermine was given orally and not when other routes of administration were used, suggesting that this process requires the interaction of the polyamine with the luminal side of the mucosa (111). On the other hand, although it has been postulated that the polyamines present in the human milk, could contribute to prevent food allergic processes in neonates, more studies appear to be necessary (112).

As commented above, antizymes and antizyme inhibitors play a relevant role in cellular polyamine uptake. However, the influence of these proteins on intestinal polyamine absorption is uncertain. Both ODC and AZ1 mRNA transcripts are abundantly expressed in small intestine (113). Treatment of the intestinal epithelial cell line IEC-6 with 10 μM of spermidine or spermine induced the synthesis of the antizyme protein negatively affecting polyamine uptake (114). In this cell line AZ1 is induced in the absence of amino acids, through a mechanism involving mTORC1 (115). Other studies using a human colon adenocarcinoma cell line (Caco-2) revealed that amino acid supplementation also modulates antizyme induction (116), whereas amino acid restriction increased spermidine uptake by Caco-2 cells, by an antizyme-independent mechanism (117). The levels of AZ mRNA have been analyzed in cells isolated from jejunal crypt-villus axis. Whereas, AZ mRNA levels were high in cells from the small intestinal crypts, the message fell to near undetectable levels in cells of the villus tip (118). However, since the expression of AZ protein was not analyzed, and it is well-known that the translation of the AZ mRNA is stimulated by polyamines, no firm conclusion about polyamine uptake though the crypt-villus axis could be established. Another uncertain question is how antizyme inhibitors may affect gut polyamine absorption. In comparison to ODC and AZ1, mRNA expression levels of AZ2, AZIN1, and AZIN2 in mouse intestine are low (113). Whereas, in normal cells AZIN1 stimulates both polyamine biosynthesis and uptake by binding to AZs, an edited form of AZIN1 with higher affinity to AZs has aroused more attention because of its contribution to carcinogenesis (119). In this regard, AZIN1 RNA editing levels were found significantly elevated in colorectal cancer tissues when compared with corresponding normal mucosa (120). It is conceivable that tissues with enhanced AZIN1 activity could accumulate higher levels of polyamines than normal ones, due to the loss of the feedback effect of polyamines by the blockade of AZ function.

## Polyamines and Gut Microbiota

**Figure 3:**
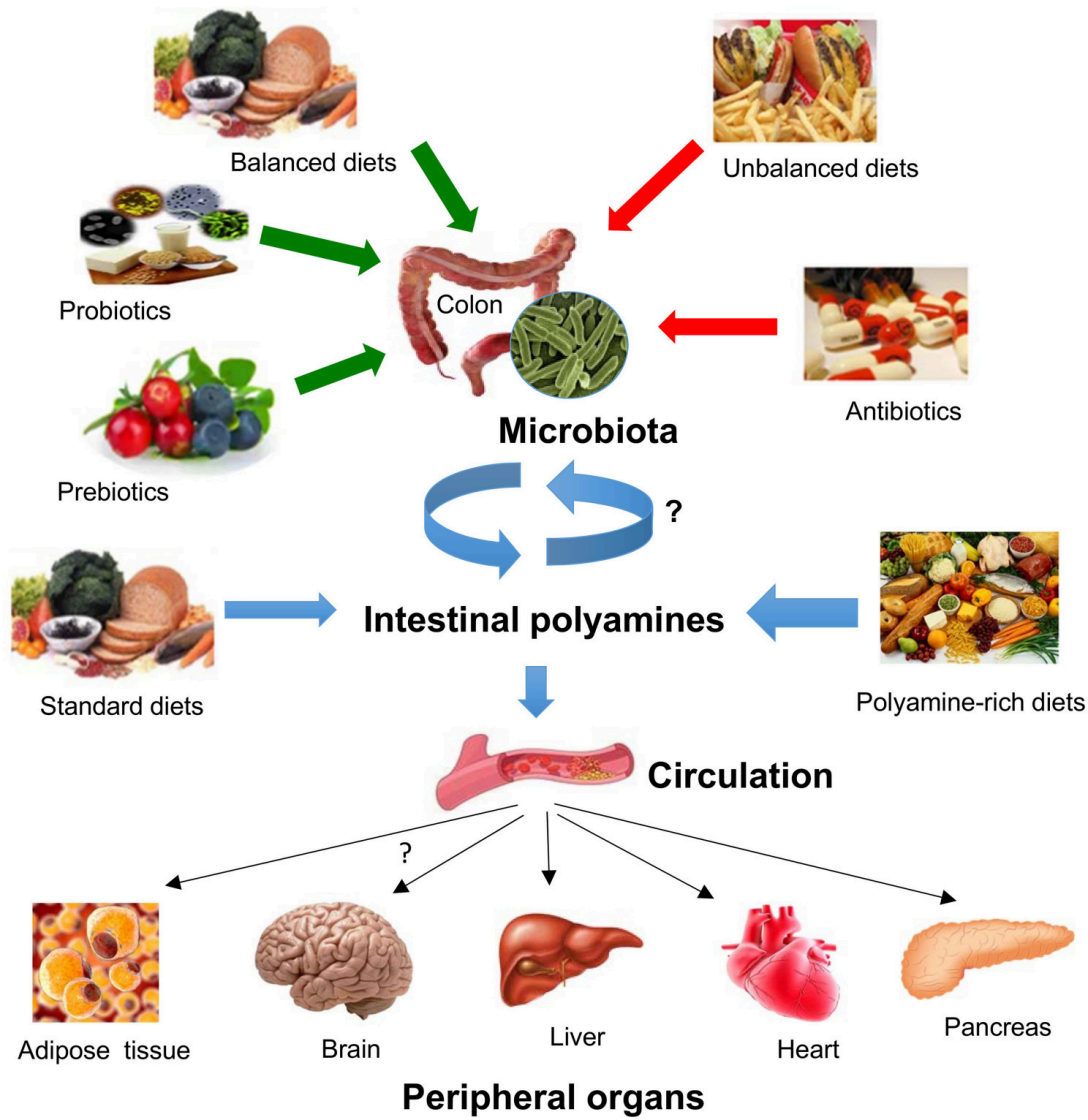
Impact of diet and gut microbiota on polyamine-mediated effects in peripheral organs. Gut microbiota composition and microbial polyamine production are affected by several conditions such as the intake of balanced/unbalanced diets, the consumption of prebiotics/probiotics, and antibiotics. Intestinal polyamines levels depends on their uptake from the gut microbiota or from different dietary conditions, which in turn it can be transported into the circulation where they can target various peripheral organs including adipose tissue, brain, liver, heart, or endocrine pancreas.

The quantity and diversity of microbial species in the gut increase longitudinally from the stomach to the colon, being the colon where the most dense and metabolically active community exist (121). The gut microbiome produces a diverse metabolite repertoire that may harm or benefit the host. These gut microbial metabolites include short-chain fatty acids, polyphenols, vitamins, tryptophan catabolites, and polyamines (122, 123). A recent study has provided data on polyamine biosynthesis and transport in the dominant human gut bacteria (124). As described above the high levels of polyamines present in the intestinal tract may be originated from the diet or produced *de novo* by host cells and intestinal bacteria (125) (Figure 3). Nevertheless, it is thought that the greatest amounts of the polyamines present in the lower parts of the intestinal tract could be mostly synthesized by intestinal microbiota. A metabolomics study in mice revealed that the intestinal luminal levels of putrescine and spermidine, but not of spermine, are mainly dependent on colonic microbiota (122). In addition, experiments in rats using different dietary interventions demonstrated that the oral administration of fermentable fibers and fructans stimulates certain intestinal microbial species (i.e., bacteroides and fusobacteria) to synthetize large amounts of polyamines in the large intestine (126–128). It is believed that polyamines synthesized by the colonic microflora are transported to the proximal gut via the portal circulation and biliary tree (129), although the contribution of colonic microflora to circulating levels of polyamines in the host is presently unknown. It is likely that part of the polyamines produced by the colonic microbiota is excreted in the feces. In this regard, it was reported that human fecal polyamine concentration may be linked to fecal microbiota (130).

Unlike the host polyamine metabolism, gut microorganisms can produce polyamines using constitutive or inducible forms of amino acid decarboxylase enzymes. Ornithine, lysine, and arginine decarboxylase activities have been detected in bacteria and archea (5). In addition, spermidine can be produced from putrescine and dcAdoMet by spermidine synthase, although some microorganisms can synthesize this polyamine from putrescine using L-aspartate β-semialdehyde as aminopropyl donor (5). This alternative biosynthetic route of spermidine is dominant in human gut microbiota (131). Arginine decarboxylation is also the dominant pathway for diamine biosynthesis in the most abundant species of the human intestinal microbiome (132). Interestingly, oral administration of arginine to mice after treatment with antibiotics did not result in increased putrescine levels in the intestinal tract, indicating that putrescine could be predominantly produced by intestinal bacteria (133). In addition, it has been demonstrated by using stable isotope labeled arginine that colonic luminal putrescine was produced by intestinal bacteria from arginine metabolism in a dose-dependent manner (133). On the other hand, the arginine deiminase pathway (an anaerobic route for arginine degradation) is common in gut commensal bacteria such as *Enterococcus, Streptococcus, Clostridium, Lactococcus*, and *Lactobacillus* species. However, whereas some of these species cannot produce putrescine because of the lack of essential polyamine biosynthetic enzymes, they acquire it from other bacterial species through a collective biosynthetic pathway catalyzed by enzymes derived from multiple bacterial species and from several extracellular intermediates (134).

The metabolism of polyamines has a central role in the regulation of systemic and mucosal adaptive immunity. Arginine is an important modulator of the immunometabolism of macrophages and T cells able to affect their effector functions (125). Thus, the metabolism of dietary arginine by the gut microbiome might have effects on the immune system (135). In this regard, mice colonized with specific microbial communities significantly contain lower amounts of arginine in their intestines compared to germ-free mice, which has been attributed to the microbiome capacity of metabolizing important amounts of arginine from the diet in the gut and, therefore, regulating the availability of arginine for the immune system (136). Furthermore, polyamines inhibit the production of inflammatory cytokines and have antioxidant effects. In fact, exogenous polyamines can decrease the release cytokines that promotes epithelial repair and barrier function. Similarly, both spermine and histamine inhibit the activation of the NLRP6 inflammasome, which is a protein complex expressed by epithelial cells that can regulate IL-18 secretion (137, 138). On the other hand, a previous study using healthy mice, described that the presence of the probiotic strain *Bifidobacterium animalis* subsp. lactis LKM512 can induce resistance to oxidative stress and promote longevity, which was dependent on enhanced microbial polyamine synthesis (139).

Dysregulated polyamine metabolism has been suggested to be involved in the tumorigenesis of colorectal cancer (CRC) and other tumors (29). A metabolomics screen comparing paired colon cancer and normal tissue samples from patients with CRC revealed that bacteria biofilm formation, even in the normal colon tissue, was associated with increased colonic epithelial cell proliferation and host-enhanced polyamine metabolism (140). In addition, bacteria-generated polyamines in biofilms may contribute to the inflammation and proliferation of colon cancer (141). Following antibiotic treatment, resected colorectal cancer tissues harbored disrupted bacterial biofilms and lowered N^1^,N^12^-diacetylspermine tissue concentrations compare to biofilm-negative colon cancer tissues, suggesting that gut microbes can induce an increase of host generated N^1^,N^12^-diacetylspermine (141). In tumor-bearing mice, the administration of antibiotics that reduces the microbial flora of the gastrointestinal tract enhanced the cytostatic effect of difluoromethyl ornithine (DFMO), a potent inhibitor of endogenous polyamine synthesis (142, 143), suggesting that reduction of bacterial polyamine synthesis together with the inhibition of the polyamine biosynthesis route should be considered as an effective antitumoral strategy.

## Polyamines in Obesity and Related Metabolic Disorders

In several transgenic mouse models, the dysregulation of the polyamine metabolism has been shown to have an impact in the regulation of the glucose, lipid, and energy homeostasis (144–149). In fact, emerging evidence suggests that increased levels of polyamines in white adipose tissue, liver or skeletal muscle could stimulates energy expenditure and confer resistance to diet-induced obesity and non-alcoholic fatty liver disease (147, 148). In addition, a number of studies using different obesity animal models have reported altered levels of polyamines in adipose tissue (150), liver (151, 152), pancreatic islets (153), and urine (154). In addition, blood polyamines have been shown to be significantly higher in obese children compared to non-obese controls (155). Remarkably, polyamine metabolism has been involved in adipogenesis (156–158), suggesting that increased polyamine levels may be implicated in adipose tissue expandability during obesity. Particularly, in 3T3-L1 preadipocytes both spermidine and spermine are essential factors at early stages of adipocyte differentiation as they modulate the expression levels of transcriptional factors implicated in the regulation of adipogenesis (18, 157, 159).

In diet-induced obesity mouse models, a high-dose daily administration of either spermidine or spermine has been shown to be an effective strategy for weight loss and improvement of the glycemic status (103, 160, 161). For instance, spermidine supplementation resulted in a significant decrease in body weight, increased glucose tolerance and insulin sensitivity and ameliorated hepatic steatosis in high-fat diet-induced obese mice (161). Furthermore, treatment with exogenous spermine has been shown to be effective to decrease body weight and fasting glucose and to improve glucose tolerance in diet-induced obese mice (160). In cultured rat adipocytes, spermine has been shown to enhance glucose transport and the conversion of glucose into triacylglycerols (162), and to increase the ability of insulin to bind to its own receptor (163). Finally, spermidine administration was able to prevent lipid accumulation and necrotic core formation in vascular smooth muscle cells through the induction of cholesterol efflux in an experimental model of atherosclerosis (164).

Despite the fact that several studies have indicated that polyamine metabolism could be dysregulated in obesity, the role of polyamines in type 2 diabetes (T2D), which is frequently associated with obesity, has been less explored. In pancreatic islets, polyamines are mainly located in the secretory granules of the β cells (52), where they have been implicated in proinsulin biosynthesis and insulin secretion (165). Islet polyamine levels diminished with age and obesity (153), suggesting that alterations in the intracellular levels of polyamines might affect β cell function. Remarkably, transgenic mice overexpressing the polyamine catabolic enzyme spermidine/spermine acetyltransferase (SSAT), which contain higher levels of putrescine and reduced levels of spermidine and spermine in the pancreatic islets, display impaired glucose-stimulated insulin secretion (146), suggesting that disturbed levels of polyamines may be involved in the physiopathology of T2D. In this regard, it has been recently reported an association between the serum levels of polyamines and T2D in a cohort of patients with metabolic syndrome (166). In particular, it was found that serum putrescine levels were significantly elevated in diabetic subjects and that correlated with glycosylated hemoglobin, and that serum spermine was closely associated with insulin levels (166). In animal models of diabetes, the polyamine biosynthetic enzyme ODC has been described to be upregulated in rat diabetic kidneys, resulting in increased levels of putrescine but not other polyamines (167).

On the other hand, exogenous administration of spermidine in rats with pharmacologically induced diabetes results in an improvement of glycaemia and a concomitant reduction of glycosylated HbA1c levels (168). Furthermore, spermine administration has been shown to be effective to protect β cells against the diabetogenic effect of alloxan (169). In diabetic rats, spermine administration did not affect hyperglycemia but improved the lipid profile and lead to a reduction in the formation of advanced glycation end-products (170). Finally, spermidine could improve endothelial dysfunction in diabetes by restoring nitric oxide production in an autophagy-dependent fashion (171).

Like polyamines, agmatine has significant metabolic effects when exogenously administered in animal models. For instance, the chronic administration of agmatine resulted in a reduction body weight gain and fat depots in rats fed a high-fat diet (172). Remarkably, this study further demonstrated that the effects of long-term agmatine consumption on body weight and adiposity could be due to the regulation of fat oxidation and gluconeogenesis (172). Additionally, recent studies have reported that exogenous agmatine administration significantly reduces atherosclerotic lesions in ApoE knockout mice (173), although the mechanism by which agmatine exerts these anti-atherosclerotic effect remains to be elucidated.

Numerous works have demonstrated that both oxidative stress and chronic inflammation are factors attributed to obesity that can contribute to the development of metabolic syndrome. Polyamines can act of modulators of oxidative stress and inflammation (174). However, the possible impact polyamines in these processes in the context of obesity have not been explored yet. Gut microbiota is another well-known factor involved in the pathogenesis of obesity and T2D. In fact, recent insight suggests that an altered composition and diversity of gut microbiota could play an important role in the development of metabolic disorders. Gut microbiota dysbiosis is characterized by metabolic endotoxemia, abnormal incretin secretion and production of bacterial metabolites that can influence energy and glucose metabolism (175). As described above polyamines can be produced by several bacterial strains in the gut, although the potential therapeutic use of polyamines derived from the gut microbiota in the treatment of metabolic disorders remains to be investigated.

## Dietary Polyamines in Aging and Cancer

Several studies have shown that the exogenous addition of polyamines, especially spermidine, increases the lifespan in yeast, nematodes, and fruit flies (101, 176). In rodents, a progressive decrease in the tissue polyamine content has been observed with age (49, 177), and the chronic administration of spermidine extends lifespan (107, 178, 179). Likewise, in animal models, supplementation with polyamines could have a beneficial effect on several age-related disorders including memory decline, neuroinflammation, and cardiovascular disease (107, 180–182). Several *in vitro* studies have shown that the administration of spermidine in cell lines of neuronal origin inhibits the process of cellular senescence (183, 184). It has been proposed that the mechanism by which polyamines produces these beneficial anti-aging effects could be through the activation of autophagy (34, 101), either through the inhibition of the activity of the acetyltransferase EP300, which is involved in the modulation of autophagic flux (185–187), or the stabilization of pro-autophagic factors such as MAP1S (179).

In addition to the exogenous supply of synthetic polyamines, several studies have shown that an increase in the concentration of gut polyamines through the administration of probiotics inhibits cellular senescence and stimulates longevity in mice (133, 139). In humans, recent epidemiological studies have associated a higher consumption of dietary spermidine with a decrease in cardiovascular events and lower mortality (107, 188). Remarkably, many food products included in the Mediterranean diet, which is known to be associated with increased longevity (189), are rich in polyamines (94, 190). However, whether a greater adherence to a Mediterranean dietary pattern increases circulating polyamine levels remains unknown.

On the other hand, in parallel with the progressive reduction of tissue polyamine levels, many works have reported alterations in the DNA methylation status associated with age (191, 192). Noteworthy, the polyamine pathway is connected to the methionine cycle through the action of AdoMetDC, which is able to decarboxylate AdoMet. In turn, AdoMet is the major donor of methyl groups for nucleic acids or other macromolecules. Thus, it has been proposed that lower spermidine and spermine levels can lead to reduced DNA methylation levels by inducing the accumulation of dcAdoMet, which it could be able to inhibit DNA methyltransferase (DNMT) activity (193). In accordance, *in vitro* experiments consisting in the pharmacological or genetic depletion of polyamine pools in human cell lines resulted in changes in DNA methylation (105, 194, 195). Moreover, abnormalities in the global DNA methylation status associated with aging can be reversed by exogenous polyamine supply (105), supporting the idea that the reduction of tissue polyamines during aging is closely related to alterations in DNA methylation. Remarkably, an abnormal DNA methylation profile has been related not only with aging, but also with other pathologies including cancer and metabolic disorders (196, 197). Interestingly, adherence to a Mediterranean dietary pattern has been described to be associated with changes in the DNA methylation status (198, 199). However, whether these effects are mediated by polyamines or other metabolites related to one carbon metabolism remain to be elucidated.

Despite the fact that emerging evidence is proposing polyamines as therapeutic option for aging-related human events including cardiovascular disease or memory decline, the pro-carcinogenic potential of polyamines should be carefully considered when polyamine-rich diets are therapeutically recommended. It is widely accepted that polyamine production is abnormally stimulated in different types of human tumors by induction of the polyamine biosynthetic pathway, which could be beneficial for tumor progression (200). Many efforts have been directed to limit the polyamine pools in the tumor by administrating chemical inhibitors of the polyamine biosynthetic enzyme ODC (i.e., DFMO), although this therapeutic option frequently has failed due to the capacity of tumor cells of replacing endogenously synthetized polyamines by extracellular polyamines from the circulation. Hence, restriction of dietary polyamines has been proposed to enhance the effects of the inhibitors of the polyamine synthesis. In CRC patients with advanced adenoma it has been proposed that the limitation of dietary polyamines could be an adjunctive strategy to avoid recurrence after 3 years of treatment using polyamine-inhibitory drugs (201). By contrast, it has been suggested that dietary polyamines might decrease the risk of CRC in postmenopausal women (202). This is supported by experiments in mice that demonstrated that polyamine intake could be an effective strategy to prevent colon tumors after 1,2-demethylhydrazine administration (105). In rats, whereas the treatment with a low spermidine diet can promote the induction of mammary tumors, a high spermidine diet may suppress tumorigenesis (203). Additionally, it has been recently reported that polyamine accumulation could have toxic effects for renal cell carcinoma and that the tumor cells avoid polyamine toxicity by inducing arginase 2 (204). Therefore, although polyamines have been classically considered as oncometabolites by its procarcinogenic actions once the tumor is formed, these new studies could indicate that dietary polyamines could be considered for cancer prevention or even for cancer treatment depending on the cancer type in a dose-dependent fashion. However, because of the insufficient number of studies performed *in vivo* with exogenously administrated polyamines in mouse cancer models, caution is necessary before considering the ingestion of polyamines as an anticancer strategy in humans.

## Concluding Remarks

Although it is well-known that the polyamine content in mammalian tissues is tightly controlled through a complex regulatory network involving the modulation of the biosynthesis, catabolism, and transport pathways, the tissue and circulating levels of polyamines are altered in a number of pathological conditions. Whereas, numerous experiments have demonstrated that inhibitors of the polyamine biosynthetic pathway drastically decrease the tissue levels of putrescine and spermidine, less is known about the effect of either the restriction or the administration of exogenous polyamines on tissue and circulating polyamine levels. Interestingly, recent studies have revealed that oral spermidine supplementation extended the lifespan and has cardioprotective effects in different animal models, but the detailed molecular mechanisms by which spermidine exert these effects are only partially understood. In humans, nutritional studies have described a relationship between spermidine-rich foods and lower cardiovascular events and reduced mortality, but in both cases, no evidence about the tissue or circulating levels of spermidine has been reported. More recently, chronic interventions of elderly subjects with controlled amounts of spermidine have revealed increased levels of this polyamine in blood after several months of treatment. Other studies have demonstrated that prolonged consumption of natto, a traditional Japanese fermented food rich in polyamines, significantly increases spermine, but not spermidine in blood of healthy young volunteers. Because of the beneficial effects of polyamine intake recently reported, it would be interesting to know how the dietary polyamine supplementation might affect polyamine levels in different organs and tissues and why this variation induces all the reported effects.

On the other hand, emerging body of evidence has revealed that in diet-induced obesity models the daily administration of high doses of polyamines (either spermidine or spermine) can reduce body weight and adiposity and can positively regulate glucose homeostasis, suggesting that exogenous polyamines can impact peripheral organs with relevant implications in lipid and glucose metabolism such as adipose tissue and liver (Figure 3). In pharmacological models of T2D, polyamines could also exert protective effects in pancreatic β cells. These effects, however, have not been proven after the oral intake of low concentrations of polyamines with demonstrated effects on longevity or heart protection. Therefore, the impact of a chronic low-dose oral polyamine intake should be performed in models of obesity or T2D before considering polyamines as therapeutic option for these metabolic disorders.

## Author Contributions

All authors: conceptualization, writing review, and editing; RP, BR-M, and MQ-O: writing original draft; RP and BR-M funding.

### Conflict of Interest Statement

The authors declare that the research was conducted in the absence of any commercial or financial relationships that could be construed as a potential conflict of interest. The reviewer MA declared a shared affiliation, with no collaboration, with several of the authors, BR-M, MQ-O, FT, to the handling editor at time of review.
